# Closed-reduction Intramedullary Screw in Fractures of the Proximal Phalanges of the Digits of the Hand: A Series of Three Cases

**DOI:** 10.1055/s-0041-1739405

**Published:** 2021-12-13

**Authors:** Arsanto Triwidodo, Hendar Nugrahadi Priambodo, Yogi Ismail Gani

**Affiliations:** 1 Departamento de Ortopedia e Traumatologia, Faculdade de Medicina, Universitas Indonesia, Koja Public Regional Hospital, Jacarta, Indonésia

**Keywords:** bone plates, bone screws, bone wires, finger phalanges, fractures, bone, fracture fixation internal

## Abstract

The most frequent skeletal injuries are hand fractures, which constitute around 20% of all fractures. Fractures of the phalanx are usual, comprising 6% of all fractures. Proximal phalanx fractures arise more often. The main goals of the care are to repair the anatomy, reduce the damage to soft tissue, and mobilize the damaged fingers as soon as stabilization of the fracture allows it. The present report is intended to examine the clinical and radiation effects of proximal phalanx fractures in patients treated with intramedullary screw fixation who underwent closed reduction. We report three consecutive cases of proximal phalanx fracture: two basal fractures and one simple shaft fracture. They were treated surgically with closed reduction with intramedullary headless compression screws. The preoperative magnitude of the angulation of the proximal phalanx averaged 30.3° (range: 13° to 42°). Two patients presented rotational deformity. The clinical findings were measured using the abbreviated version of the Disabilities of the Arm, Shoulder and Hand (Quick-DASH) questionnaire, and the range of motion and functional results were assessed. Complications were observed over a span of at least 3 months. The patients displayed complete active flexion-extension proximal interphalangeal joint and flexion-extension metacarpophalangeal joint without rotative deformity. The scores on the QuickDASH were satisfactory (mean: 2.3; range: 0 to 4.5). No secondary surgeries were performed, and complications were not observed. Intramedullary fixation of proximal phalanx fractures with cannulated tension screws has been shown to be a safe operation, resulting in outstanding functional performance and an early recovery. The fracture is minimized and properly consolidated by the patients.

## Introduction

Hand fractures are known to be the most frequent skeletal injuries, accounting for ∼ 20% of all fractures. 1 2 Phalanx fractures represent 6% of all fractures. The primary objectives of the treatment are to recover the anatomy, to prevent unnecessary soft-tissue injury, and to enable early mobilization of the injured site. 3

The general solution to phalangeal fractures is as follows: buddy taping or splint, for undisplaced extra-articular fractures, surgical treatment of displaced articular parts, rotational malalignment, or > 15° or 6 mm of shortening. 4 The surgical procedures may include closed reduction and pinning or open reduction and fixation. Closed reduction and internal fixation provide interfragmentary compression and the option of earlier mobilization, while open reduction and external fixation only provide interfragmentary compression. 5

We report three cases of proximal phalanx fractures of the hand submitted to closed reduction with the fixation of intramedullarys and early mobilization.

## Case Report

**Fig. 1:**
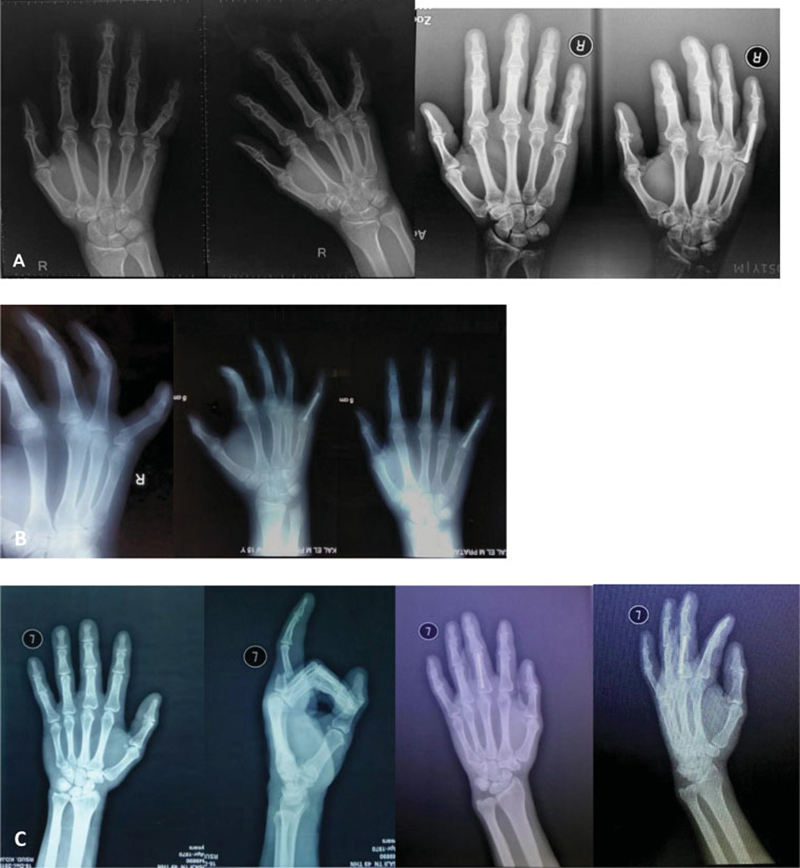
Preoperative (left) and postoperative (right) radiographic evaluations: ( **A** ) Case 1. ( **B** ) Case 2. ( **C** ) Case 3.

We report three cases of proximal phalanx fracture: two transverse fractures at the base of the phalanx and one simple transverse shaft fracture. All patients were right-handed, and one patient injured the non-dominant hand. An X-ray of the hand was performed ( Fig. 1 ). All fractures were closed and not associated with neurovascular injury. Every patient was treated surgically with closed reduction with the fixation of intramedullary screws.

## Operative Technique

The surgeries were performed by a senior orthopedist in a single academic hospital using an identical technique in each of them. The diameter of the intramedullary canal of each patient was evaluated on a profile radiograph. In every operation, screws with a diameter of 2.2 mm were used. The antegrade technique and intra-articular technique were used on all fingers in these surgeries.

**Fig. 2:**
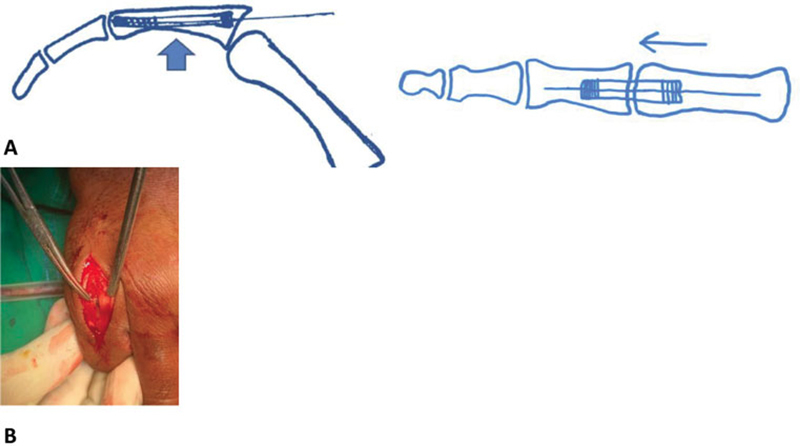
( **A** ) Schematic anterograde intraarticular technique; sagittal and axial views ( **B** ). The incision at the dorsal side of the metacarpophalangeal joint.

The reduction of the fractures was performed under fluoroscopy and corrected by tractioning and manipulating the finger in case of rotational deformity. A 2-cm incision and dorsal capsulotomy were performed to expose the intraarticular metacarpophalangeal (MCP) joint as shown in Fig. 2 . A guide wire was inserted into the dorsal central portion of the base of the proximal phalanx, with the MCP joint with ∼ 60° of flexion. The base of the finger was softly moved dorsally so the wire could be placed.

Once the guide wire was in the proper place and a satisfactory reduction was achieved, a headless cannulated screw was inserted into the metaphysis of the proximal phalanx. The guide wire was removed. The length of the screw was measured using the radiograph and tested under fluoroscopy before insertion. Special precaution was taken to ensure that the base of the screw did not protrude, and that the top of the screw hit the end of the medullary canal. The complete range of motion (ROM) of the MCP and proximal interphalangeal (PIP) joints was tested before skin closure. Postoperatively, a hand-therapy regimen was prescribed to all patients.

## Evaluation

All patients were evaluated at minimum 3-month follow-up. At the latest follow up, the score on the Quick Disabilities of the Arm, Shoulder and Hand (QuickDASH) questionnaire was obtained, and any persisting symptoms were noted. A clinical examination was performed to evaluate any deformity, and the active ROM of the MCP and PIP joints was measured using a goniometer.

Todos os pacientes alcançaram completa ADM ativa e ativo-assistida das articulações MCF e IFP na primeira semana do pós-operatório, e demonstraram uma ADM de flexo-extensão ativa total e indolor dessas articulaçõe sem qualquer deformidade (latência prologada/má rotação) no último acompanhamento. A flexão média da articulação MCF foi de 85° (variação: 80° a 90°), e a flexão média da articulação IFP, 99° (variação: 90° a 110°). A pontuação média no QuickDASH foi de 2,3 (variação: 0 a 4,5). Não houve queixas quanto à sensibilidade nas articulações MCF e IFP, no sítio da fratura, ou casos de refratura. Nenhum dos pacientes apresentou complicações que exigissem tratamento adicional ou cirurgia secundária. O quadro clínico e os resultados estão resumidos na Tabela 1 .

**Tabela 1:** Quadro clínico e resultados

Caso	Gênero	Idade	Mão acometida	Característica da fratura	Lesão associada			Resultado clínico					
						Angulação dorsal do ápice		Deformidade rotacional		ADM pós-op. da flexão da articulação MCF	ADM pós-op.da flexão da articulação IFP	Complicação	Pontuação no QuickDASH
						Pré-op.	Pós-op.	Pré-op.	Pós-op.				
1	M	51	D	Falange proximal da 5ª base	N	42	0	N	N	85°	101°	N	0
2	M	18	D	Falange proximal da 5ª base	N	35	0	S	N	87°	99°	N	2,3
3	M	49	N-D	Falange proximal da 3ª diáfise	N	14	0	S	N	84°	96°	N	4,5

Abreviaturas: ADM, amplitude de movimento; D, dominante; IFP, interfalangeana proximal; M, masculino; MCF, metacarpofalangeana; N, não; N-D, não dominante; Pós-op., pós-operatório; Pré-op., pré-operatório; QuickDASH, versão abreviada do questionário de Disfunções do Braço, Ombro e Mão (Quick Disabilities of the Arm, Shoulder and Hand, em inglês); S, sim.

All patients achieved full active and active-assisted ROM of the MCP and PIP joints within the first postoperative week, and demonstrated painless full active flexion-extension ROM of these joints without any deformity (extension lag/malrotation) at the latest follow up. The mean flexion of the MCP joint was of 85° (range: 80° to 90°), and the mean flexion of the PIP joint was of 99° (range: 90° to 110°). The mean score on the Quick-DASH was of 2.3 (range: 0 to 4.5). No complaints regarding tenderness at MCP or PIP joints or at the fracture site, or re-fracture, were reported. No patients had complications that required further treatment or secondary surgery. Table 1 presents a summary of the results and clinical picture.

**Table 1:** Clinical picture and results

Case	Gender	Age	Injured hand	Characteristic of the fracture	Associated injury			Clinical result					
						Apex dorsal angulation		Rotational deformity		Postop. MCP joint flexion ROM	Postop. PIP joint flexion ROM	Complication	QuickDASH score
**Preop.**	**Post op.**	**Pre op.**	**Post op.**
1	M	51	D	5th-base proximal phalanx	N	42	0	N	N	85°	101°	N	0
2	M	18	D	5th-base proximal phalanx	N	35	0	Y	N	87°	99°	N	2.3
3	M	49	N-D	3rd-shaft proximal phalanx	N	14	0	Y	N	84°	96°	N	4.5

Abbreviations: D, dominant; M, male; MCP, metacarpophalangeal; N, no; N-D, non-dominant; PIP, proximal interphalangeal; Postop., postoperative; Preop., preoperative; QuickDASH, Quick Disabilities of the Arm, Shoulder and Hand questionnaire; ROM, range of motion; Y, yes.

## Discussion

The goal of the search for less intrusive techniques is to reduce the risks, thus enabling early mobilization. 2 Most phalangeal fractures can be managed non-operatively. However, in the cases herein reported, the fractures were relatively unstable, requiring surgery with intramedullary screws to achieve an excellent result. 3 4

An unstable finger fracture that is not fit for surgery also has an uncertain optimum treatment. Plate fixation helps offer stabilization for an early recovery, although the clinical outcomes have been inconsistent. 5 Minimally invasive-techniques can offer viable alternatives to the fixation of phalangeal fractures to soft tissue. Internal or external fixation may be used. 6 Both solutions have their own pitfalls linked to unplanned hardware withdrawal, immobilization, and incomplete fixation. 7

A less intrusive method of treating dysfunctional extra-articular fractures of the proximal phalanx is successful and secure. Analyses show promising results following intramedullary screw fixation for metacarpals and phalanges with reduced complications. 8 9 10 There were no malunions or rotational deformities, no illness or dynamic regional pain condition, and no migration of any screws after 3 months follow-up. The ROM of the finger was perfect. The results revealed that 75% of the ROM of the wrist was recovered in two weeks, and complete ROM was attained at the final follow-up.

Com a fixação de parafuso de compressão intramedular nas fraturas da falange proximal, os pacientes demonstraram um excelente desempenho funcional, e tiveram uma recuperação precoce e completa. As fraturas foram suficientemente reduzidas e consolidadas. As complicações podem ser difíceis de tratar, e a não consolidação pode acarretar prejuízos para o resultado funcional.

With the fixation of proximal phalanx fractures with intramedullary compression screws, the patients exhibited outstanding functional performance, an experienced an early and full recovery. The fractures were sufficiently minimized and consolidated. The complications may be challenging to handle, and malunions can lead to a poor functional result.
