# Supplementary material for: Closed-reduction Intramedullary Screw in Fractures of the Proximal Phalanges of the Digits of the Hand: A Series of Three Cases
Source: Rev Bras Ortop (Sao Paulo). 2021 Dec 13;59(Suppl 1):e34–8. doi: 10.1055/s-0041-1739405 (PMC11254437; doi:10.1055/s-0041-1739405)
Supplement: Related texts

# Research article: Parafuso intramedular de redução fechada em fraturas das falanges proximais dos dedos da mão: Uma série de três casos

**Keywords:** falanges dos dedos da mão, fios ortopédicos, fixação de fratura interna, fraturas ósseas, parafusos ósseos, placas ósseas

## Abstract

As lesões esqueléticas mais frequentes são as fraturas que ocorrem nas mãos, que representam aproximadamente 20% de todas as fraturas. As mais comuns são as fraturas falangeanas, que abrangem 6% de todas as fraturas. As fraturas da falange proximal aparecem com mais frequência. O principal objetivo do atendimento é corrigir a anatomia, reduzir os danos aos tecidos moles, e mobilizar os dedos atingidos assim que a estabilização da fratura permitir. Este relato tem como objetivo examinar os efeitos clínicos e da radiação nas fraturas da falange proximal, em pacientes infectados com a fixação de parafuso intramedular que foram submetidos a redução fechada. Este é o relato de três casos consecutivos de fratura da falange: duas fraturas da base e uma fratura simples da diáfise. Todos foram submetidos a redução fechada com parafuso intramedular de compressão sem cabeça. A magnitude pré-operatória da angulação da falange proximal foi em média 30,3° (variação de 13° a 42°). Dois pacientes apresentaram deformidade rotacional. Os achados clínicos foram medidos com a versão abreviada do questionário de Disfunções do Braço, Ombro e Mão (Quick Disabilities of the Arm, Shoulder and Hand, QuickDash, em inglês), em que foram avaliados a amplitude de movimento e os resultados funcionais. Complicações foram observadas em um período mínimo de 3 meses. Os pacientes apresentavam flexo-extensão ativa total na articulação interfalangiana proximal e na articulação metacarpofalangiana de flexo-extensão sem deformidade rotacional. A pontuação no Quick-DASH foi satisfatória (média: 2,3; variação: 0 a 4,5). Não houve cirurgias secundárias, e não se observaram complicações. A fixação intramedular das fraturas da falange proximal com o parafuso de tensão canulado tem se mostrado uma cirurgia segura, com excelente desempenho funcional e recuperação precoce. A fratura é minimizada e consolidada adequadamente pelos pacientes.

## Introdução

As fraturas das mãos são reconhecidamente as lesões esqueléticas mais frequentes, e são responsáveis por ∼ 20% de todas as fraturas. 1 2 As fraturas falangeanas representam 6% de todas as fraturas. Os objetivos primários do tratamento são: corrigir a anatomia, evitar lesões desnecessárias dos tecidos moles, e permitir a mobilização precoce do sítio da lesão. 3

A solução geral para as fraturas falangeanas é a seguinte: usar a técnica de solidarização ou tala, fraturas extra-articulares saudáveis, tratamento cirúrgico das partes articulares deslocadas, desalinhamento rotacional, ou encurtamento > 15° ou 6 mm. 4 Os procedimentos cirúrgicos podem incluir redução fechada e pinagem ou redução aberta e fixação. A redução fechada e a fixação interna fornecem compressão interfragmentária e uma alternativa de mobilização mais precoce, ao passo que a redução aberta e a fixação externa fornecem apenas compressão interfragmentária. 5

Relatamos três casos de fraturas da falange proximal da mão submetidas ao método de redução fechada com a fixação de parafuso intramedular e à mobilização precoce.

## Relatos de Casos

Relatamos três casos de fratura da falange proximal: duas fraturas transversais da base da falange, e uma fratura transversal simples da diáfise. Todos os pacientes eram destros, e um deles apresentava lesão na mão não dominante. Foi realizada uma avaliação radiográfica da mão ( Fig. 1 ). Todas as fraturas foram fechadas, e não houve associação com lesão neurovascular. Todos os pacientes foram submetidos a redução fechada com a fixação de parafuso intramedular.

**Fig. 1:** Avaliações radiológicas pré-operatória (esquerda) e pós-operatória (direita): ( **A** ) Caso 1. ( **B** ) Caso 2. ( **C** ) Caso 3.

## Técnica Cirúrgica

As cirurgias foram realizadas por um ortopedista sênior em um único hospital universitário, usando uma técnica idêntica em todas elas. O diâmetro do canal intramedular de cada paciente foi avaliado por meio de radiografia na incidência de perfil. Usamos um parafuso de 2,2 mm de diâmetro em cada cirurgia. Foram utilizadas as técnicas anterógrada e a intra-articular em todos os dedos.

A redução das fraturas foi realizada sob fluoroscopia, sendo corrigida por tração e manipulação do dedo em caso de deformidade rotacional. Foi realizada uma incisão de 2 cm, seguida de uma capsulotomia dorsal, a fim de expor a metacarpofalangeana (MCF) intra-articular, conforme mostrado na Fig. 2 . Um fio guia foi inserido na porção dorsal central da base da falange proximal, com a articulação MCF com aproximadamente 60° de flexão. A base do dedo foi suavemente movida dorsalmente para que o fio pudesse ser colocado.

**Fig. 2:** ( **A** ) Esquema da técnica intra-articular anterógrada; incidências sagital e axial ( **B** ) da incisão do lado dorsal da articulação metacarpofalangeana.

Uma vez que o fio guia estava no devido lugar e tendo sido alcançada uma redução satisfatória, um parafuso canulado sem cabeça foi inserido na metáfise da falange proximal. O fio guia foi removido. O comprimento do parafuso foi medido por meio de radiografia, e testado mediante fluoroscopia antes da inserção. Tomou-se cuidado especial para garantir que a base do parafuso não se projetasse, e para que a extremidade superior do parafuso atingisse a extremidade do canal medular. A amplitude de movimento (ADM) completa das articulações MCF e interfalangeana proximal (IFP) foi testada antes do fechamento da pele. No pós-operatório, prescreveu-se um regime fisioterapêutico da mão em todos os pacientes.

## Avaliação

Todos os pacientes foram avaliados com um acompanhamento mínimo de 3 meses. No último acompanhamento, foi realizada uma avaliação com com a versão abreviada do questionário de Disfunções do Braço, Ombro e Mão (Quick Disabilities of the Arm, Shoulder and Hand, QuickDash, em inglês), em que foram observados quaisquer sintomas persistentes. Foi realizado um exame clínico para avaliar qualquer deformidade, e a ADM ativa das articulações MCF e IFP foi medida com um goniômetro.

## Discussão

Buscar técnicas menos invasivas tem como objetivo a redução dos riscos, permitindo assim a mobilização precoce do dedo. 2 A maioria das fraturas falangeanas podem ser tratadas de forma não cirúrgica. No entanto, nos casos aqui descritos, as fraturas eram relativamente instáveis, e necessitavam de cirurgia com parafuso intramedular para a obtenção de um resultado excelente. 3 4

Em casos de fratura instável do dedo para a qual a cirurgia não é adequada, a operação ideal é incerta. A fixação de placas ajuda a oferecer estabilização para a recuperação precoce, embora os resultados clínicos sejam inconsistentes. 5 Técnicas minimamente invasivas podem oferecer alternativas viáveis para a fixação das fraturas falangeanas nos tecidos moles. Pode ser usada uma fixação interna ou externa. 6 Ambas as soluções têm suas próprias armadilhas, associadas à retirada não planejada de *hardware* , à imobilização, e à fixação incompleta. 7

O emprego de um método menos invasivo de tratamento das fraturas extra-articulares disfuncionais da falange proximal é mais bem-sucedido e seguro. Análises mostram resultados promissores após a fixação do parafuso intramedular para metacarpos e falanges, com um número reduzido de complicações. 8 9 10 Após três meses de acompanhamento, não houve fraturas, deformidades rotacionais, nenhuma doença, condição de dor regional dinâmica, e nenhuma migração de qualquer parafuso. A ADM do dedo foi perfeita. Os resultados revelaram que 75% da ADM do punho foram recuperados em 2 semanas, sendo que a ADM completa foi alcançada no acompanhamento final.
